# Expression of a-Tocopherol-Associated protein (TAP) is associated with clinical outcome in breast cancer patients

**DOI:** 10.1186/s12907-015-0021-5

**Published:** 2015-12-09

**Authors:** Xi Wang, Brian Z. Ring, Robert S. Seitz, Douglas T. Ross, Kirsten Woolf, Rodney A. Beck, David G. Hicks, Shuyuan Yeh

**Affiliations:** Department of Pathology, University of Rochester Medical Center, Rochester, NY 14642 USA; Institute for Genomic and Personalized Medicine, School of Life Science and Technology, Huazhong University of Science and Technology, Wuhan, China; Insight Genetics Inc., Nashville, TN USA; CardioDx, Inc., Redwood City, CA USA; Conversant Biologics, Huntsville, AL USA

**Keywords:** Breast cancer, α-Tocopherol-associated protein (TAP), Vitamin E

## Abstract

**Background:**

The role of vitamin E in breast cancer prevention and treatment has been widely investigated, and the different tocopherols that comprise this nutrient have been shown to have divergent associations with cancer outcome. Our previous studies have shown that α-Tocopherol-associated protein (TAP), a vitamin E binding protein, may function as a tumor suppressor-like factor in breast carcinogenesis. The current study addresses the association of TAP expression with breast cancer clinical outcomes.

**Methods:**

Immunohistochemical stain for TAP was applied to a tissue microarray from a breast cancer cohort consisting of 271 patients with a median follow-up time of 5.2 years. The expression of TAP in tumor cells was compared with patient’s clinical outcome at 5 years after diagnosis. The potential role of TAP in predicting outcome was also assessed in clinically relevant subsets of the cohort. In addition, we compared TAP expression and Oncotype DX scores in an independent breast cancer cohort consisting of 71 cases.

**Results:**

We demonstrate that the expression of TAP was differentially expressed within the breast cancer cohort, and that ER+/PR ± tumors were more likely to exhibit TAP expression. TAP expression was associated with an overall lower recurrence rate and a better 5-year survival rate. This association was primarily in patients with ER+ tumors; exploratory analysis showed that this association was strongest in patients with node-positive tumors and was independent of stage and treatment with chemotherapy. TAP expression in ER/PR negative or triple negative tumors had no association with clinical outcome. In addition, we did not observe an association between TAP expression and Oncotype DX recurrence score.

**Conclusions:**

The significant positive association we found for α-Tocopherol-associated protein with outcome in breast cancer may help to better define and explain studies addressing α-tocopherol’s association with cancer risk and outcome. Additionally, further studies to validate and extend these findings may allow TAP to serve as a breast-specific prognostic marker in breast cancer patients, especially in those patients with ER+ tumors.

## Background

Breast cancer is the most common malignant tumor in women worldwide, comprising 16 % of all female cancers. Epidemiological studies have shown that vitamin E has a potential utility in the prevention and treatment of human malignancies, including breast cancer [1–3]. However clinical trials on the effectiveness of dietary supplementation with vitamin E or *α-*tocopherol, the principle and most active vitamin E isoform in human plasma, as an aid in the prevention of cancer have not produced evidence of a consistent association with decreased cancer occurrence [4, 5]. The inconstancies between epidemiological studies and intervention trials may be due to differing roles for the tocopherols that comprise vitamin E or unexplained genetic diversity in the study populations affecting how they utilized vitamin E.

Our previous studies have shown that *α-*Tocopherol-associated protein (TAP), a vitamin E binding protein [6], is selectively expressed in human breast, prostate, liver and brain tissue and its expression can be evaluated by immunohistochemical staining [7, 8]. We have shown that, while TAP can facilitate vitamin E retention in cancer cells and promote vitamin E-mediated anti-proliferation effects, it can also act as a tumor suppressor-like protein in a vitamin E-independent fashion. Overexpression of TAP in prostate cancer cells was shown to suppress cell growth; and a TAP siRNA knockdown in a prostate cell line led to increased cell growth [7]. In human breast, we identified that TAP is typically co-expressed with ER in sporadic normal/benign luminal cells in terminal ductal lobular units, and that TAP showed decreased expression in 57 % of invasive breast carcinomas, including 46 % of ER and PR positive carcinomas, and 80 % of high grade carcinomas [9]. Another study has shown that TAP mRNA level is negatively associated with tumor stage and lymph node status in breast cancer [10]. TAP expression therefore may be a candidate for a marker of less aggressive breast carcinoma.

Despite the advances in multidisciplinary treatment, breast cancer remains the second most common cause of death related to cancer in women. In addition to the routine pathologic characteristics of breast cancer, such as tumor size, grade, vascular invasion, lymph node metastasis, ER/PR/Her2 status etc., genes which may have an association with tumor biology and help in predicting recurrence, therapeutic response and survival have been studied widely. Currently there are at least nine gene expression signatures showing some correlation with certain clinical breast cancer outcomes [11–16]. The genes included in these panels are diverse but largely related to cell cycle regulation and proliferation, the ER pathway, and to a lesser degree, the immune system. Although these gene panels have similar outcomes performance, they exhibit a large degree of discordance in the assignment of a particular breast tumor to a specific prognostic group [11]. More accurate prognostic predictive gene signatures will depend on better understanding of the genes specifically involved in breast cancer carcinogenesis.

To further investigate if TAP expression is associated with clinical outcome in breast carcinomas, we studied TAP expression in a breast cancer cohort of 271 patients diagnosed with invasive breast carcinomas with median follow up time of 5.2 years. In addition, in an independent cohort of 71 breast cancer cases, we compared TAP expression with the Oncotype DX recurrence score to determine if there was a correlation between TAP expression and this clinically available multigene prognostic/predictive assay.

## Methods

A tissue micro-array comprising 288 patient samples from a primary invasive breast cancer cohort from the Clearview Cancer Institute (CCI, AL, U.S.A.), consisting of all available patient samples collected from 1990 to 2001, was constructed. The patient average age was 58.9 (range 26–89), with an average tumor size 2.21 cm (range 0.2–8.0) and a median follow up time of 5.2 years. Ninety eight tumors were stage 1, 141 stage 2, and 33 stage 3. One hundred fifty four patients had negative lymph nodes, while 118 patients had positive lymph node(s). One hundred thirty two patients had received chemotherapy plus hormonal therapy, while 156 were untreated or treated with hormonal therapy only. No patients had been treated with Herceptin. Thirty six tumors were grade 1, 106 were grade 2 and 93 were grade 3. Within this cohort, 271 patients had complete follow-up and TAP expression data. The composition of this set of patients is as shown in Table 1.

**Table 1:** Cohort characteristics. *P* value for difference between proportion of clinical characteristic within TAP positive and negative patients determined with a two-proportion z-test, except for age and tumor size for which a *t*-test was employed

	All cases		TAP negative		TAP positive		
	No. of patients	%	No. of patients	%	No. of patients	%	*P* value
Total	271		183		88		
Grade							
1	34	12.5	20	10.9	14	15.9	0.33
2	105	38.7	59	32.2	46	52.3	0.02
3	92	33.9	78	42.6	14	15.9	0.03
Unknown	40	14.8	26	14.2	14	15.9	nd
Stage							
I	97	35.8	60	32.8	37	42	0.18
II	137	50.6	94	51.4	43	48.9	0.39
III	31	11.4	23	12.6	8	9.1	0.4
Unknown	6	2.2	6	3.3		0	nd
Age (avg, range)							
	58.1 (26–89)		56.9 (26–87)		62.6 (35–89)		<0.001
T							
T1	138	50.9	82	44.8	56	63.6	0.01
T2	101	37.3	74	40.4	27	30.7	0.19
T3	14	5.2	12	6.6	2	2.3	0.41
T4	8	3	6	3.3	2	2.3	0.47
Unknown	10	3.7	9	4.9	1	1.1	nd
N							
N0	150	55.4	97	53	53	60.2	0.2
N1	108	39.9	74	40.4	34	38.6	0.43
N2	6	2.2	5	2.7	1	1.1	0.46
Unknown	7	2.6	7	3.8		0	nd
M							
M0	258	99.2	172	98.9	86	100	1
M1	2	0.8	2	1.1		0	nd
Unknown	11		9		2		nd
Tumor size (avg, cm)							
	2.09		2.39		1.83		<0.001
Received chemotherapy							
no	133	49.1	75	41	58	65.9	1
yes	130	48	101	55.2	29	33	1
Unknown	8	3	7	3.8	1	1.1	nd
ER							
ER-	65	24	58	31.7	7	8	0.1
ER+	197	72.7	120	65.6	77	87.5	0
Unknown	9	3.3	5	2.7	4	4.5	nd
HER2							
HER2-	116	42.8	85	46.4	31	35.2	1
HER2+	72	26.6	43	23.5	29	33	1
Unknown	83	30.6	55	30.1	28	31.8	nd
Hormone therapy							
No	77	26.7	57	28.8	20	22.2	0.28
yes	187	64.9	120	60.6	67	74.4	0.03
Unknown	24	8.3	21	10.6	3	3.3	nd

An independent cohort of 71 invasive breast carcinomas for which Oncotype DX (Genomic Health, Inc.) recurrence scores had been determined were identified from the files of the Department of Pathology and Laboratory Medicine at Strong Memorial Hospital (Rochester, NY). All cases were hormone receptor positive, Her2 negative, and lymph node negative. A representative whole tissue section was cut from each tumor.

This study was approved by the Huntsville Hospital Institutional Review Committee. Archived tumor samples were provided by the Clearview Cancer Institute of Huntsville Alabama and corresponding anonymized patient data was provided via an institutional review board–approved database. An IRB exemption for the use of the tissue samples was granted by the Huntsville Hospital Institutional Review Committee as all patient data were a) anonymized, b) consent was unnecessary, and c) only excess tissue was used.

**Fig. 1:**
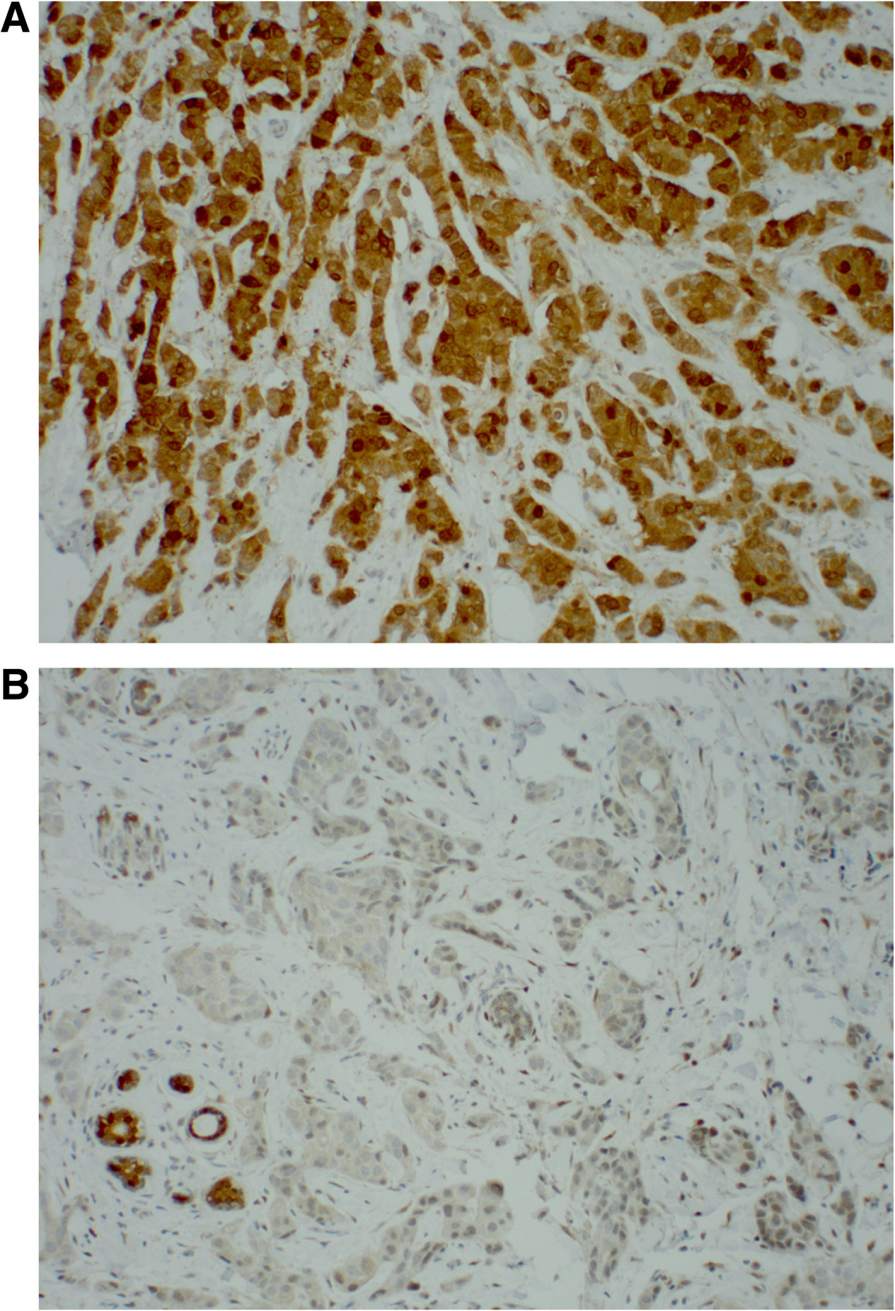
Invasive ductal carcinoma showing TAP staining positive (**a**), and negative with the positive internal control of normal/benign TDLU (**b**)

Tissue arrays were processed as previously described [17]. TAP antibody was generated in house as previously published. Immunohistochemical staining for TAP was performed on tissue micro-array and whole tissue sections with the method we described previously [8]. TAP expression was classified as positive or negative, with positive expression defined as any cytoplasmic and/or nuclear staining (Fig. 1). Commercial antibodies for ER, PR, and HER2 were stained by a commercial service (US Labs Inc). ER and PR were considered positive if at least 1 % of the cells examined exhibited any nuclear staining, and HER2 was scored positive when intense membrane staining in more than 10 % of invasive tumor cells was observed.

In assessing association with outcome, the likelihood ratio test was used in univariate analyses, and the Wald test for multivariate models. All *p* values are presented as two sided, with a value of less than 0.05 being considered significant.

## Results

**Fig. 2:**
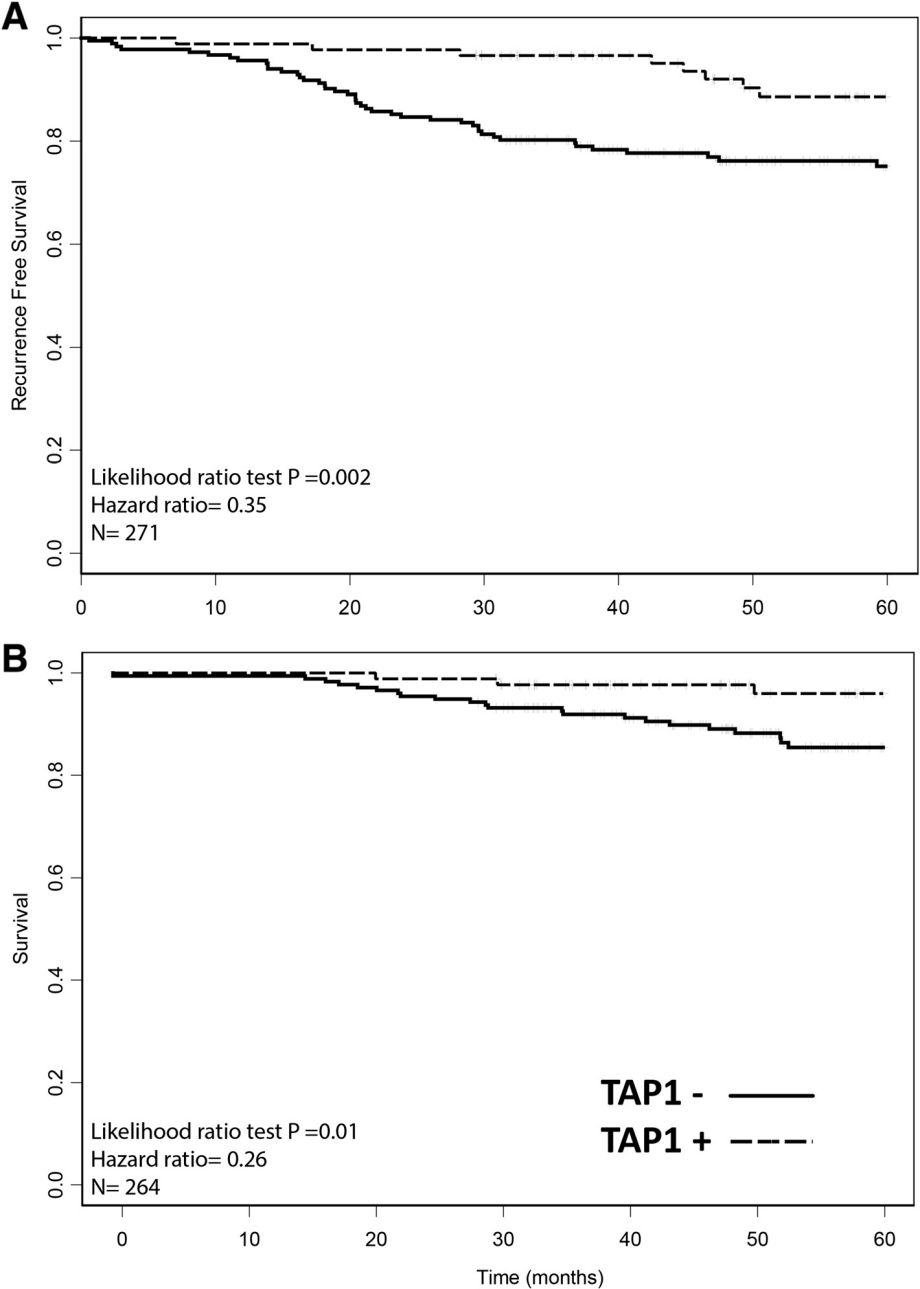
Patients with TAP-positive tumors had a lower 5-year recurrence rate (**a**) and better 5-year survival rate (**b**) than the patients with TAP-negative tumors. TAP-positive tumors are shown via a dotted line, TAP-negative tumors via a solid line

**Fig. 3:**
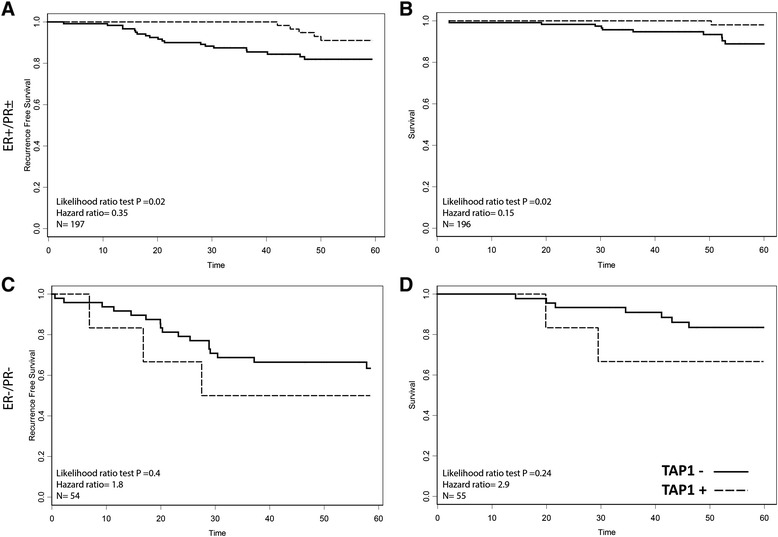
In patients with ER+/PR ± positive tumors, TAP positivity was associated with a better 5 year recurrence (**a**) and survival (**b**). In patients with hormone negative (ER-/PR-) tumors TAP showed a non-significant negative association with 5 year recurrence (**c**) and survival (**d**)

**Fig. 4:**
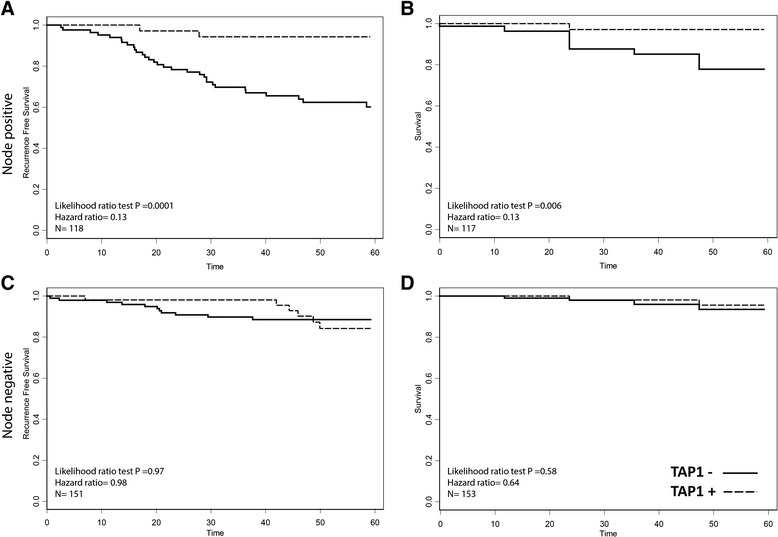
The association between TAP expression and better prognosis was even stronger in node-positive patients in 5 year recurrence (**a**) and survival (**b**), but was not significant in node-negative patients in 5 year recurrence (**c**) or survival (**d**)

In the CCI breast cohort of 271 breast carcinoma samples, we observed positive TAP staining in 88 (32 %) and negative staining in 183 (68 %) tumors. Consistent with our previous findings, we found that ER+/PR ± tumors are more likely to exhibit TAP expression than hormone negative tumors (Table 2). Overall, patients with TAP-positive tumors had a lower 5-year recurrence rate (*N* = 271, *p* = 0.002) and better 5-year survival rate (*N* = 264, *p* = 0.010) (Fig. 2a, b). This positive association with outcome was conserved in all ER-positive tumors, regardless of PR status (5 year recurrence, HR = 0.35, *p* = 0.02, 5 year survival, HR = 0.141, *p* = 0.014) (Fig. 3a, b). This association was also significant at 10 years post diagnosis (10 year survival HR = 0.21, *p* = 0.0024; 10 year recurrence HR = 0.55, *p* = 0.023). Looking further at the clinically relevant ER+/PR±/Her2- patients, significant associations were also observed (5 year recurrence, HR = 0.17, *p* = 0.035, 5 year survival, HR = <0.001, *p* = 0.007, *N* = 81). TAP was not prognostic of 5 year recurrence in ER+/HER2+ cases (HR = 0.38 *p* = 0.22, *N* = 57), ER-/HER2-(HR < 0.01, *p* = 0.23, *N* = 32), or ER-/HER2+ cases (HR = 4.1 *p* = 0.28, *N* = 15). Tumors negative for ER and PR had a non-significant association with 5-year recurrence and survival (Fig. 3c, d). In triple negative patients the association remained non-significant, though low patient numbers (*N* = 25) makes it difficult to draw conclusions from this subset analysis. Exploratory analysis showed that the association with outcome was even stronger in node-positive patients (5-year recurrence: *N* = 118, *p* = 0.0001; 5-year survival: *N* = 114, *p* = 0.0036), but it was not significant in node-negative patients (5-year recurrence: *N* = 151 and *p* = 0.98; 5-year survival: *N* = 150 and *p* = 0.72) (Fig. 4a-d). Within PR subsets (ER+/PR- and ER+/PR+), TAP did not have a significant association with recurrence (*p* = 0.102 and 0.098, respectively), though the hazard ratio remained low. Furthermore, TAP was independent of PR when assessed as a multivariable model (data not shown).

**Table 2:** TAP and hormone receptor status, patient counts. *P* value for difference between proportion of TAP positive and negative patients determined with a two-proportion z-test

	TAP-	TAP+	*P* value
Hormone Receptor Negative (ER & PR-)	48	6	<0.001
ER-	58	7	<0.001
Hormone Receptor Positive (ER or PR+)	127	78	<0.001
ER+	120	77	0.001
HR positive/ HER2-	56	29	0.003
HR positive/ HER2+	31	28	0.35

In an exploratory multivariate analysis of TAP status and common clinical variables, TAP was independent of age, stage, hormonal therapy and chemotherapy status with 5 year recurrence as the outcome measurement in all patients (Table 3). TAP was not independent of grade, which suggests that grade and TAP were measuring a shared aspect of cell biology. Indeed, there was a significant negative association between grade and TAP (p < 0.0001 via chi-square). TAP positive tumors were more likely to represent low grade tumors compared to TAP negative tumors. In patients with ER+/PR ± tumors, TAP expression was independent of stage and age in predicting 5 year recurrence; while in patients with ER+/PR±/Her2- tumors, TAP was not independent of stage (5 year recurrence HR = 0.18, *p* = 0.110). However, only 81 patients were included in this subset. When looking at 5 year survival, TAP expression was independent of age and stage, but not chemotherapy status or tumor grade.

**Table 3:** Association of TAP and other clinical variables with outcome using Cox proportional hazard regression. TAP, chemotherapy, grade, stage and age are shown as individual and multivariable models. Variables with a significant association with survival or recurrence are shown in bold

	5 year survival			5 year recurrence			5 year recurrence, ER+/PR±		
	HR	p	N	HR	p	N	HR	p	N
**TAP**	0.26 (0.08, 0.87)	0.01	264	0.35 (0.16, 0.74)	0.002	271	0.35 (0.13, 0.92)	0.02	197
Hormone therapy	0.54 (0.25, 1.2)	0.12	262	0.68 (0.38, 1.2)	0.21	261	1.8 (0.55, 6.2)	0.28	192
**Chemotherapy**	4.34 (1.63, 11.6)	0.001	262	2.51 (1.37, 4.6)	0.002	263	2.2 (1, 4.84)	0.05	193
**Grade**	4.44 (1.74, 11.3)	<0.001	230	2.3 (1.34, 3.93)	0.001	231	2.5 (1.24, 5.05)	0.007	175
**Stage**	3.35 (1.82, 6.16)	<0.001	264	2.71 (1.76, 4.17)	<0.001	265	2.66 (1.47, 4.83)	0.001	195
Age	0.98 (0.95, 1.01)	0.193	264	0.98 (0.97, 1)	0.054	265	0.97 (0.94, 1)	0.058	195
**TAP**	0.28 (0.08, 0.95)	0.04	264	0.38 (0.18, 0.81)	0.012	265	0.37 (0.14, 0.98)	0.045	195
**Stage**	3.25 (1.76, 6)	<0.001		2.63 (1.71, 4.06)	<0.001		2.57 (1.41, 4.69)	0.002	
TAP	0.55 (0.16, 1.93)	0.35	230	0.54 (0.23, 1.24)	0.15	231	0.51 (0.19, 1.39)	0.19	174
**Grade**	3.93 (1.52, 10.18)	0.005		2.05 (1.19, 3.52)	0.01		2.3 (1.15, 4.59)	0.018	
**TAP**	0.28 (0.08, 0.94)	0.039	264	0.38 (0.18, 0.82)	0.014	265	0.37 (0.14, 0.98)	0.045	195
Age	0.99 (0.96, 1.02)	0.37		0.98 (0.96, 1.01)	0.16		0.97 (0.94, 1.01)	0.097	
**TAP**	0.27 (0.08, 0.90)	0.032	262	0.37 (0.17, 0.78)	0.0093	261	0.35 (0.13,0.93)	0.036	192
Hormone therapy	0.58 (0.27, 1.3)	0.17		0.73 (0.41, 1.3)	0.3		1.77 (0.53, 5.9)	0.35	
**TAP**	0.34 (0.1, 1.14)	0.081	262	0.42 (0.2, 0.91)	0.027	263	0.38 (0.14, 1.01)	0.052	193
**Chemotherapy**	3.69 (1.37, 9.92)	0.01		2.16 (1.17, 4.01)	0.014		1.94 (0.87, 4.3)	0.1	
**TAP**	0.33 (0.1, 1.13)	0.078	262	0.41 (0.19, 0.89)	0.023	263	0.38 (0.14, 1.03)	0.057	193
**Stage**	2.77 (1.42, 5.4)	0.003		2.44 (1.53, 3.9)	0		2.42 (1.27, 4.61)	0.007	
Chemotherapy	2.21 (0.79, 6.17)	0.13		1.35 (0.7, 2.59)	0.37		1.23 (0.53, 2.85)	0.63	
**TAP**	0.32 (0.1, 1.1)	0.07	262	0.41 (0.19, 0.89)	0.024	263	0.39 (0.15, 1.06)	0.065	193
**Stage**	2.8 (1.43, 5.47)	0.003		2.44 (1.53, 3.9)	0		2.53 (1.34, 4.76)	0.004	
Chemotherapy	2.98 (0.89, 10)	0.077		1.34 (0.61, 2.94)	0.47		0.85 (0.31, 2.32)	0.75	
Age	1.02 (0.98, 1.06)	0.35		1 (0.97, 1.03)	0.98		0.97 (0.94, 1.01)	0.19	

In the study of tumors with a known Oncotype DX recurrence score, TAP was positive in 47 of the 71 tumors (66.2 %), with 29 of 43 (67.4 %) in a low risk group, 16 of 24 (66.7 %) in an intermediate risk group, and 2 of 4 (50 %) in a high risk group (Table 4). There was no association observed between TAP expression and Oncotype DX recurrence risk using a Fisher’s exact test (*P* = 0.60).

**Table 4:** Comparison of TAP positivity and OncoTypeDx Recurrence scores

Recurrence Score	TAP +	TAP -	Total
1	29	14	43
2	16	8	24
3	2	2	4
Total	47	24	71

The limitation of the study includes relatively small sample size in the CCI breast cohort and Oncotype DX cohort. Ki67 labeling index data and the status of vascular invasion were not available for analysis.

## Discussion

Genes involved in cell proliferation have been shown to comprise a major component of many of the available gene expression signatures used to predict clinical outcome in breast cancer patients [11–16]. TAP has also been implicated in the control of cellular proliferation and other aspects of tumor growth. Researchers demonstrated that vitamin E derivatives can inhibit cell proliferation and colony formation of breast cancer cell lines, and induce apoptosis of the tumor cells [18–21]. As a vitamin E binding protein, TAP can promote vitamin E retention and thus increase its concentration in cells. Our previous studies have demonstrated that TAP can promote vitamin E-induced inhibition of tumor cell proliferation and also regulate tumor cell growth in a vitamin E-independent fashion [7]. We also showed that TAP is selectively expressed in normal/benign breast luminal epithelium, but not in many other organ systems [8]. TAP expression is down-regulated at the mRNA and protein levels in several human breast cancer cell lines and in human breast carcinomas compared to a nonmalignant cell line and to normal/benign breast tissue. We extended these observations in current study, which showed that TAP expression is associated with an overall more favorable outcome, both in terms of tumor recurrence and survival rate in breast cancer patients. These findings, taken together, suggest that TAP is a regulator of cell proliferation that can affect breast carcinogenesis and tumor prognosis.

Breast cancer is a heterogeneous group of diseases. The biological features and clinical behavior of individual tumors are frequently different, even within the morphologically low grade, ER+/PR+/Her2- subtype, which is generally considered to be a group with a better prognosis. It is important to identify novel markers which can predict clinical outcomes in this group. We have reported that TAP is co-expressed with ER in the normal/benign breast luminal epithelium of terminal ductal lobular units, where breast carcinogenesis is most likely to be initiated, and that TAP is down regulated in 46 % of ER and PR positive breast carcinomas, indicating that the loss of TAP expression may be associated with the process of hormonal carcinogenesis [9]. Here we have demonstrated that TAP expression in ER+/PR±/Her2- tumors is associated with a significantly better 5-year recurrence free and survival rate. This finding could be clinically significant in terms of further predicting tumor behaviors of ER+/PR±/Her2- subtype breast carcinomas. Currently, there are few breast carcinogenesis-related oncogenes and tumor suppressor genes that are implicated in the clinical treatment and prognosis. Her2 and p53, the most widely evaluated cancer-related genes, are only altered in approximately 20-25 % of breast carcinomas, primarily in ER/PR negative tumors [22]. In contrast, TAP is altered in 57 % of breast carcinomas, including 46 % ER/PR+ tumors, thus may serve as a useful complement to existing biomarkers.

OncotypeDX has been used to guide clinical approaches for ER-positive, lymph node-negative breast cancer patients. Even though TAP expression is associated with better clinical outcome in ER-positive tumors, we did not identify any association between TAP expression and OncotypeDX recurrence scores in the additional cohort. This may suggest that TAP expression is associated with a different aspect(s) of tumor biology than is OncotypeDX and may be a useful complement in predicting patient outcome and tumor subclassification. Further study with a larger population of ER+ tumors is needed to help validate these findings.

Several studies have demonstrated a protective effect of vitamin E on the occurrence of several cancers (for review see [3]). However clinical trials have found little support for dietary supplementation [23]. The disparity of these results could be due to vitamin E not being cancer preventive at the supra-nutritional level, significant roles for other vitamin E isoforms, such as γ- and δ-tocopherols [24], or genetic diversity among dietary intervention trial participants contributing to unrecognized heterogeneity in how they utilized vitamin E. Our study found considerable heterogeneity among the tumors in expression of TAP, the vitamin E binding protein, and its significant association with cancer progression. This finding suggests a possible role for TAP in the interplay between vitamin E and cancer progression. Stratification of trial participation by TAP expression may be an interesting and important aspect for the elucidation of how dietary vitamin E supplementation may affect cancer risk and prognosis.

## Conclusions

In summary, we demonstrated that TAP, as a proliferation-related gene in breast carcinogenesis, is associated with a better 5-year clinical outcome, particularly in node-positive and ER+ breast cancer patients. TAP may serve as a prognostic marker, especially in those patients with ER+ low grade breast cancers, and may also serve to stratify studies assessing the role and utility of vitamin E or α-tocopherol supplementation for the prevention of cancer.

## References

[1] Wada S. Cancer preventive effects of vitamin E, vol. 13, 2011/04/07 edn. 10.2174/138920112798868656 21466429

[2] Kline K, Yu W, Sanders BG (2004). Vitamin E and breast cancer. J Nutr.

[3] Ju J, Picinich SC, Yang Z, Zhao Y, Suh N, Kong AN (2010). Cancer-preventive activities of tocopherols and tocotrienols. Carcinogenesis.

[4] Klein EA, Thompson IM, Tangen CM, Crowley JJ, Lucia MS, Goodman PJ (2011). Vitamin E and the risk of prostate cancer: the Selenium and Vitamin E Cancer Prevention Trial (SELECT). JAMA.

[5] Lee IM, Cook NR, Gaziano JM, Gordon D, Ridker PM, Manson JE (2005). Vitamin E in the primary prevention of cardiovascular disease and cancer: the Women’s Health Study: a randomized controlled trial. JAMA.

[6] Zimmer S, Stocker A, Sarbolouki MN, Spycher SE, Sassoon J, Azzi A (2000). A novel human tocopherol-associated protein: cloning, in vitro expression, and characterization. J Biol Chem.

[7] Ni J, Wen X, Yao J, Chang HC, Yin Y, Zhang M (2005). Tocopherol-associated protein suppresses prostate cancer cell growth by inhibition of the phosphoinositide 3-kinase pathway. Cancer Res.

[8] Wang X, Ni J, Hsu CL, Johnykutty S, Tang P, Ho YS (2009). Reduced expression of tocopherol-associated protein (TAP/Sec14L2) in human breast cancer. Cancer Invest.

[9] Johnykutty S, Tang P, Zhao H, Hicks DG, Yeh S, Wang X (2009). Dual expression of alpha-tocopherol-associated protein and estrogen receptor in normal/benign human breast luminal cells and the downregulation of alpha-tocopherol-associated protein in estrogen-receptor-positive breast carcinomas. Mod Pathol.

[10] Tam KW, Ho CT, Lee WJ, Tu SH, Huang CS, Chen CS (2013). Alteration of alpha-tocopherol-associated protein (TAP) expression in human breast epithelial cells during breast cancer development. Food Chem.

[11] Reyal F, van Vliet MH, Armstrong NJ, Horlings HM, de Visser KE, Kok M (2008). A comprehensive analysis of prognostic signatures reveals the high predictive capacity of the proliferation, immune response and RNA splicing modules in breast cancer. Breast Cancer Res.

[12] Paik S, Shak S, Tang G, Kim C, Baker J, Cronin M (2004). A multigene assay to predict recurrence of tamoxifen-treated, node-negative breast cancer. N Engl J Med.

[13] Paik S, Tang G, Shak S, Kim C, Baker J, Kim W (2006). Gene expression and benefit of chemotherapy in women with node-negative, estrogen receptor-positive breast cancer. J Clin Oncol.

[14] Teschendorff AE, Naderi A, Barbosa-Morais NL, Pinder SE, Ellis IO, Aparicio S (2006). A consensus prognostic gene expression classifier for ER positive breast cancer. Genome Biol.

[15] van de Vijver MJ, He YD, van’t Veer LJ, Dai H, Hart AA, Voskuil DW (2002). A gene-expression signature as a predictor of survival in breast cancer. N Engl J Med.

[16] Wang Y, Klijn JG, Zhang Y, Sieuwerts AM, Look MP, Yang F (2005). Gene-expression profiles to predict distant metastasis of lymph-node-negative primary breast cancer. Lancet.

[17] Ring BZ, Seitz RS, Beck R, Shasteen WJ, Tarr SM, Cheang MC (2006). Novel prognostic immunohistochemical biomarker panel for estrogen receptor-positive breast cancer. J Clin Oncol.

[18] Anderson K, Simmons-Menchaca M, Lawson KA, Atkinson J, Sanders BG, Kline K (2004). Differential response of human ovarian cancer cells to induction of apoptosis by vitamin E Succinate and vitamin E analogue, alpha-TEA. Cancer Res.

[19] Malafa MP, Neitzel LT (2000). Vitamin E succinate promotes breast cancer tumor dormancy. J Surg Res.

[20] Neuzil J, Weber T, Gellert N, Weber C (2001). Selective cancer cell killing by alpha-tocopheryl succinate. Br J Cancer.

[21] Yu W, Simmons-Menchaca M, Gapor A, Sanders BG, Kline K (1999). Induction of apoptosis in human breast cancer cells by tocopherols and tocotrienols. Nutr Cancer.

[22] Hamilton A, Piccart M (2000). The contribution of molecular markers to the prediction of response in the treatment of breast cancer: a review of the literature on HER-2, p53 and BCL-2. Ann Oncol.

[23] Stratton J, Godwin M (2011). The effect of supplemental vitamins and minerals on the development of prostate cancer: a systematic review and meta-analysis. Fam Pract.

[24] Lu G, Xiao H, Li GX, Picinich SC, Chen YK, Liu A (2010). A gamma-tocopherol-rich mixture of tocopherols inhibits chemically induced lung tumorigenesis in A/J mice and xenograft tumor growth. Carcinogenesis.

